# Supplementary material for: All-at-once RNA folding with 3D motif prediction framed by evolutionary information
Source: Nat Methods. 2025 Oct 3;22(10):2094–106. doi: 10.1038/s41592-025-02833-w (PMC12510878; doi:10.1038/s41592-025-02833-w)
Supplement: Supplementary file 3 — Source code to run CaCoFold-R3D. Data and results. [file 41592_2025_2833_MOESM3_ESM.gz › supplemental_material/Tables/Table_S2.pdf]

RFAM SEED ALIGNMENTS
4,178 Families

Type	Motif	# Motif instances with cov support (all)	# Rfam families with motif with cov support (all)	SSU eukarya with support (all)	LSU eukarya with support (all)
HL	GNRA_tetraloop	170 (214)	101 (132)	4 (4)	6 (7)
IL	K_turn	68 (83)	54 (68)	2 (2)	2 (2)
BL	Docking_elbow	59 (94)	55 (83)	2 (2)	2 (5)
J3	J3_groupII	52 (57)	40 (43)	1 (1)	1 (1)
HL	UNCG_tetraloop	48 (78)	42 (69)	1 (1)	0 (0)
J3	J3_typeA	46 (49)	36 (39)	4 (4)	4 (4)
BL	AA_bulge	45 (101)	34 (81)	1 (1)	2 (2)
IL	pK_turn	45 (82)	41 (75)	0 (0)	0 (0)
J3	J3_typeC	43 (45)	42 (43)	0 (0)	1 (1)
J4	J4_U1RNA	40 (44)	29 (31)	0 (0)	3 (4)
IL	UAA_GAN	40 (50)	23 (33)	0 (0)	5 (5)
IL	G_bulge	37 (52)	30 (39)	0 (0)	1 (1)
IL	K_turn_b	35 (50)	32 (47)	0 (1)	1 (1)
J4	J4_HCV_IRES	33 (36)	25 (28)	3 (3)	1 (1)
IL	Hook_turn	32 (49)	28 (45)	0 (0)	3 (3)
IL	Tandem_GA	29 (37)	19 (25)	2 (3)	3 (3)
BS	LoopE_a	28 (33)	15 (20)	1 (1)	4 (4)
BS	T_loop	27 (33)	25 (30)	1 (1)	2 (2)
J4	J4_manA	26 (27)	24 (25)	0 (0)	2 (2)
IL	C_loop	26 (42)	25 (40)	1 (1)	0 (0)
BS	CsrA_motif.rev	26 (30)	23 (27)	2 (2)	1 (1)
HL	UNAC_tetraloop	26 (48)	26 (47)	0 (0)	1 (1)
BS	CRC_binding	26 (34)	18 (22)	5 (5)	0 (0)
J4	J4_tRNA	25 (26)	19 (20)	3 (3)	3 (3)
HL	T_loop_tetraloop	24 (43)	20 (34)	1 (3)	0 (0)
BS	LoopE_b.rev	24 (25)	18 (18)	1 (1)	1 (1)
BS	LoopE_b	21 (22)	15 (16)	1 (1)	2 (2)
BL	bulged_G	20 (43)	18 (37)	0 (0)	0 (0)
BS	T_loop.rev	20 (26)	17 (22)	1 (1)	3 (3)
HL	CUYG_tetraloop	20 (44)	20 (43)	0 (0)	0 (0)
HL	AA_5SrRNA	19 (19)	18 (18)	0 (0)	0 (0)
HL	ANYA_tetraloop	18 (39)	16 (35)	0 (0)	1 (1)
IL	J4a/4b	16 (37)	16 (35)	0 (0)	1 (1)
J3	J3_typeB	16 (16)	14 (14)	2 (2)	0 (0)
J3	J3_hammerhead	16 (27)	16 (26)	0 (0)	1 (1)
HL	L8_RNaseP_bact_a	16 (29)	14 (26)	0 (0)	0 (1)
BS	LoopE_a.rev	16 (21)	13 (17)	0 (0)	1 (1)
HL	U_turn	15 (24)	14 (23)	0 (0)	0 (1)
HL	GANC_tetraloop	15 (28)	14 (26)	0 (0)	2 (2)
BS	CRC_binding.rev	15 (18)	8 (11)	6 (6)	0 (0)
BS	CsrA_motif	15 (18)	13 (16)	0 (0)	0 (1)
J3	J3_TPP	14 (19)	12 (17)	0 (0)	0 (1)
HL	YGNN_tetraloop	13 (27)	12 (26)	0 (0)	0 (0)
IL	GUA_GG_RRE	10 (13)	10 (12)	0 (0)	0 (0)
HL	CsrA_binding	10 (29)	7 (14)	0 (1)	0 (0)
HL	GAGUA_pentaloop	10 (22)	9 (21)	0 (0)	0 (0)
IL	S_domain	10 (21)	10 (20)	0 (0)	0 (0)
IL	Loop_E	9 (11)	9 (10)	0 (0)	1 (2)
IL	Twisted_up	9 (31)	9 (30)	0 (0)	0 (0)
J3	J3_purine	7 (11)	7 (11)	0 (0)	0 (0)
IL	GAAA_Tetraloop_receptor	7 (17)	7 (17)	0 (0)	0 (0)
HL	U6_loop	7 (18)	7 (18)	0 (0)	0 (1)
IL	J4/5_IL	6 (13)	6 (13)	0 (1)	0 (0)
HL	Sarcin_ricin_loop	5 (7)	5 (7)	0 (0)	1 (1)
IL	G_bulge_Das	5 (11)	5 (11)	0 (0)	0 (1)
IL	Docking_elbow_IL	0 (1)	0 (1)	0 (0)	0 (0)

Rfam SEEDS
4,178 alignments

	# Motif instances with cov support (all)	# Rfam families with motif with cov support (all)	SSU eukarya with support (all)	LSU eukarya with support (all)
**3D Motifs all**	1460 (2124)	591 (822)	45 (51)	62 (74)
**Nested helices**	3877 (15395)	1721 (4168)	66 (89)	102 (152)
**PK + higher order**	504 (532)	246 (246)	1 (2)	2 (4)

Rfam CONTROLS
4,178 shuffled alignments

**3D Motifs all**	121 (290)	106 (208)	0 (0)	0 (1)
**Nested helices**	733 (14146)	676 (4149)	0 (34)	0 (75)
**PK + higher order**	175 (188)	102 (102)	0 (0)	0 (0)

Table S2. **RNA 3D motifs found in Rfam.** Results of running CaCoFold-R3D for all seed alignments in the database of structured RNAs Rfam. Results show RNA 3D motif occurrences with covariation support where at least one of the closing helices has at least one covarying base pair, and in parenthesis the total set of predictions. For the other structural elements (nested helices, pseudoknots and other higher order base pair interactions), having covariation support means that the element includes at least one significantly covarying pair of residues. In parenthesis we show the total number of predictions. The control shuffled alignments were obtained by randomizing the residues within each alignment column independently from each other. In these control alignments, covariation between columns is scrambled, but the base composition per column (thus the possible 3D motif identity) remains mostly intact. Results of running CaCoFold-R3D on the Rfam seed alignments were obtained using the command line `R-scape -s --cacofold --r3d Rfam.seed`. The control experiments were obtained using the command line `R-scape -s --cacofold --r3d --vshuffle Rfam.seed`.
